# Effective Antiviral Application of Antisense in Plants by Exploiting Accessible Sites in the Target RNA

**DOI:** 10.3390/ijms242417153

**Published:** 2023-12-05

**Authors:** Cornelia Gruber, Torsten Gursinsky, Selma Gago-Zachert, Vitantonio Pantaleo, Sven-Erik Behrens

**Affiliations:** 1 Institute of Biochemistry and Biotechnology, Section Microbial Biotechnology, Martin Luther University Halle-Wittenberg, D-06120 Halle (Saale), Germany; cor.gru@googlemail.com (C.G.); torsten.gursinsky@biochemtech.uni-halle.de (T.G.); selma.gago-zachert@bct.uni-halle.de (S.G.-Z.); 2 Institute for Sustainable Plant Protection, Department of Biology, Agricultural and Food Sciences National Research Council, Bari Unit, I-70126 Bari, Italy; vitantonio.pantaleo@cnr.it

**Keywords:** siRNA, antisense, ASO, RNase H, AGO, RNA silencing, RNAi

## Abstract

Antisense oligodeoxynucleotides (ASOs) have long been used to selectively inhibit or modulate gene expression at the RNA level, and some ASOs are approved for clinical use. However, the practicability of antisense technologies remains limited by the difficulty of reliably predicting the sites accessible to ASOs in complex folded RNAs. Recently, we applied a plant-based method that reproduces RNA-induced RNA silencing in vitro to reliably identify sites in target RNAs that are accessible to small interfering RNA (siRNA)-guided Argonaute endonucleases. Here, we show that this method is also suitable for identifying ASOs that are effective in DNA-induced RNA silencing by RNases H. We show that ASOs identified in this way that target a viral genome are comparably effective in protecting plants from infection as siRNAs with the corresponding sequence. The antiviral activity of the ASOs could be further enhanced by chemical modification. This led to two important conclusions: siRNAs and ASOs that can effectively knock down complex RNA molecules can be identified using the same approach, and ASOs optimized in this way could find application in crop protection. The technology developed here could be useful not only for effective RNA silencing in plants but also in other organisms.

## 1. Introduction

Targeted regulation of gene expression at the post-transcriptional level, i.e., at the level of mRNA or viral genomic RNA, can be accomplished in several ways. The most commonly used technologies are RNA interference (RNAi) and antisense oligodeoxynucleotide (ASO or A-ODN)-based methods. RNAi originally evolved in cells to combat viral infections and transposable elements, and it is a major component of the native immune response of plants, nematodes, oomycetes, fungi, and insects. RNAi is triggered by double-stranded (ds) RNAs or double-stranded elements in target RNAs. Such dsRNAs may be, for example, replication products of positive-strand RNA viruses: positive-strand RNA viruses include important pathogens in plants as well as animals and humans; their genomes function as mRNAs and are replicated via negative-strand RNA intermediates [1,2]. Cellular RNase III-like endonucleases, named Dicers or Dicer-like proteins (DCLs), recognize dsRNAs and process them into 20–24 nt-long double-stranded small interfering RNAs (siRNAs) [3,4]. Argonaute (AGO) proteins belonging to the group of RNase H-like endonucleases then incorporate one siRNA strand, which guides AGO and other proteins of RNA-induced silencing complexes (RISC) to RNA targets through base pairing. The targets generally correspond to RNA molecules originally recognized by Dicers/DCLs. They are inactivated (silenced) by AGO/RISC-mediated endonucleolytic cleavage or translational inhibition [5,6]. The RNAi mechanism is also present in mammalian cells, where it acts mainly with micro RNAs (miRNAs); however, it can also be routinely used for post-transcriptional modulation of gene expression by siRNAs [7]. Thus, siRNAs have recently been approved for various therapeutic approaches [8,9].

Significantly longer than RNAi, ASO-based antisense technologies have been used to disrupt or modulate gene expression on the RNA level [10,11]. Hybridization of readily synthesized 8–25 nucleotide-long single-stranded ASOs with the complementary sequence of a target nucleic acid can affect its function in several ways. In practice, two main technical variants are used: In the steric blocking mechanism, binding of the ASO to the target inhibits processes such as splicing, translation, and/or replication. In the degradation pathway, the binding of an ASO to a target RNA leads to the recruitment of RNase H enzymes, which then hydrolyze the target endonucleolytically at the DNA/RNA hybrid [12]. The applicability and potential of antisense technology became clear with the first approval of ASO-based therapeutics for humans in 1998 [13].

Many experimental and therapeutic indications of ASOs aim to silence target RNAs through the degradation mechanism catalyzed by RNases H [14,15]. In mammalian cells, where an RNase H is present in the nucleus and, to a lesser extent, in the cytoplasm, there is evidence that the enzyme is involved in cellular DNA replication and repair [12,15]. ASOs to be used for the formation of DNA/RNA hybrids and RNases H catalyzed degradation must contain at least five consecutive nt that hybridize with the target, with longer complementary sequences significantly increasing specificity. The 5′-phosphate/3′-hydroxyl products formed during cleavage are degraded by exonucleases, with the rate of degradation depending on the chemistry of the ASO but also on the nature of the targeted RNA [11,12].

As outlined, both AGO/RISC and RNase H endonucleases target RNAs via short nucleic acid guides, i.e., the siRNA guide strand and the ASO, respectively. Therefore, a basic requirement for endonuclease-mediated silencing is the accessibility of the target sequence to which the respective nucleic acids (NAs) can hybridize. However, most RNA molecules, such as mRNAs or the genomes of RNA viruses, are folded in a complex way and, under natural conditions, are additionally masked, e.g., by RNA-binding proteins, ribosomes, or viral replication machinery [16,17]. It is, therefore, obvious that the more structured and inaccessible a target RNA is, the lower the probability of association of the guiding NA and, thus, of the active endonucleases [18,19]. Indeed, in target RNAs spanning several kilobases, very few regions, hereafter referred to as “a-sites” (accessible sites), are accessible to nucleic acid/endonuclease complexes. This relationship has been demonstrated for siRNA/RISC, e.g., by Gago-Zachert et al. [20], and for ASO/RNase H, e.g., by Vickers and Crooke [21].

A reliable prediction of a-sites in complex RNA molecules has so far proved difficult or nigh impossible. RNA structures can be determined experimentally in aqueous solution and even in the cell (see, for example, [22,23]). However, these approaches are technically demanding, and they only provide RNA structures under precisely defined conditions. If conditions change, and this is constantly the case during the activity of an RNA in the cell, simply because of the dynamic interactions of RNA-binding proteins, experimental structure determination is not useful for a-site prediction. Although there are a number of approaches to predict RNA structures and siRNA or ASO binding sites (see, for example, http://crdd.osdd.net/servers/virsirnapred, accessed on 30 November 2023; [24,25,26,27]), these are usually subject to uncertainties (reviewed in [8,11]) and can only be verified by elaborate empirical studies in target cells or organisms. Errors in predicting a-sites in complex, folded target RNAs are an important reason for recurrent efficiency problems in silencing approaches.

In previous studies, we developed a straightforward experimental screening method that allows reliable identification and functional characterization of siRNAs, here called *e*siRNAs, which induce AGO/RISC-mediated RNAi with high efficiency (*e* stands for efficient) on a target RNA of choice [20] (https://www.sciencemag.org/news/2019/08/new-medicine-could-vaccinate-plants-against-devastating-viruses, accessed on 30 November 2023). Here, we show that ASOs derived in their sequence from *e*siRNAs are comparably effective in RNase H-mediated antisense silencing and can be similarly used in an antiviral approach. Our observations suggest that the screening method used, hereafter referred to as “*e*NA-screen”, is generally capable of identifying a-sites in complex RNA molecules that can be targeted by different types of nucleic acid-directed endonucleases. Our data also indicate that optimized ASOs can be used in alternative plant protection concepts.

## 2. Results

### 2.1. Nicotiana tabacum Cells Contain Different Types of RNase H Activities 

**Figure 1:**
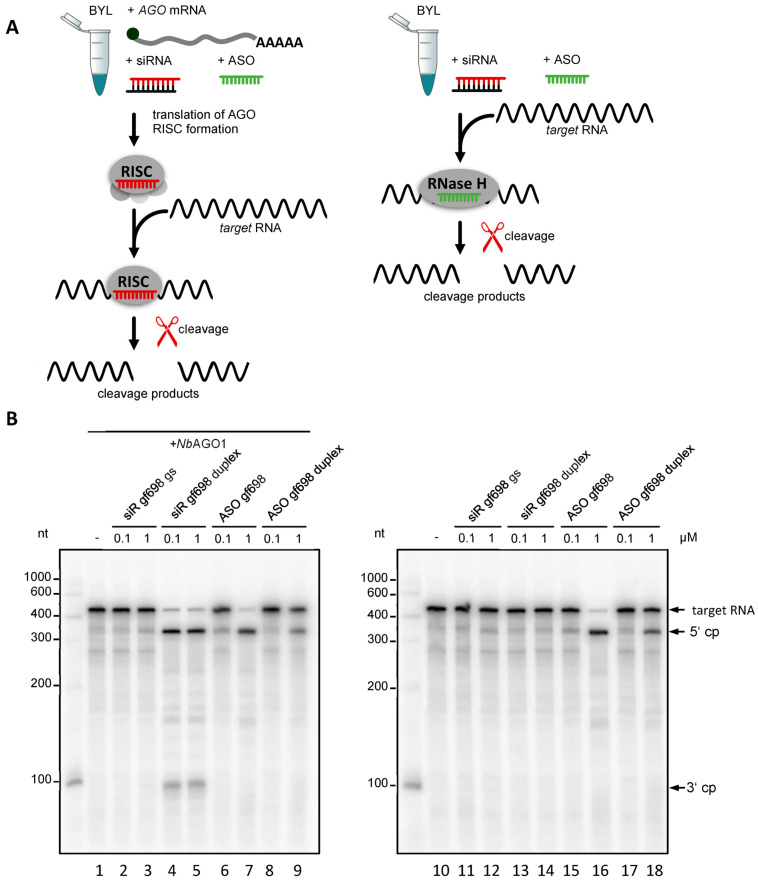
DNA-directed cleavage of a target RNA in *Nicotiana tabacum* BYL. (**A**) Schematic representation of cleavage assays that were performed in the presence or absence of heterologous AGO protein. RNA-directed cleavage was performed with the siRNA duplex (siR gf698) targeting GFP mRNA or with the corresponding siRNA guide strand (gs) indicated in red. DNA-directed cleavage of the target RNA was performed with an antisense oligonucleotide (ASO gf698) whose sequence corresponds to that of the siRNA guide strand (indicated in green) or, alternatively, with a double-stranded oligonucleotide (ASO duplex) whose sequence corresponds to that of the siRNA duplex (not shown). (**B**) The assays shown were performed in the presence (**left**) or absence (right) of *N. benthamiana* AGO1 protein generated by in vitro translation of the corresponding mRNA (see **A**) in BYL and with different concentrations of the added nucleic acids (0.1 and 1 µM, respectively). Cleavage of the ^32^P-labeled template was monitored by denaturing PAGE [30]; the cleavage products (cp) are indicated.

Previous studies have established cytoplasmic extracts of culture-grown *N. tabacum* cells, so-called BY-2 lysates (BYL), as versatile tools for various applications, which support the translation of proteins as well as the replication of positive-strand viral RNAs in vitro [28,29]. Most importantly, BYL also recapitulates the primary RNA-silencing process: i.e., exogenously added dsRNAs are processed by extract-endogenous DCLs [30], and, with an in vitro-translated AGO protein of choice, active RISC can be assembled with siRNAs (Figure 1A). The applied siRNAs can either be endogenously generated by DCLs from dsRNAs or natural target RNAs containing dsRNA-elements or added exogenously as synthetic molecules [30,31]. The activity of the in vitro-generated RISC can be tested in a “slicer-” or “cleavage assay”, where AGO-mediated cleavage (slicing) of a target RNA is detected [30,31,32,33] (Figure 1A). The BYL system was also applied to establish the above-mentioned screening method, which allows reliable identification and functional characterization of *e*siRNAs that induce AGO/RISC-mediated RNAi with high efficiency on a chosen target RNA: In a first step, a pool of siRNAs is generated from the target RNA by BYL-endogenous DCLs. Subsequently, siRNAs are identified by RNA seq that bind with high affinity to an in vitro-translated AGO protein. Finally, the fraction of these AGO-binding siRNAs that trigger particularly efficient hydrolysis of the target RNA in the cleavage assay is determined [20].

The initial experiments of this study investigated whether BYL, besides RNA-directed hydrolysis, also supports DNA-directed hydrolysis of target RNAs. To test this, we used a segment of the mRNA of green fluorescent protein (GFP) as a model target RNA and the 21 nt-long siRNA duplex, siR gf698, directed against an accessible site of the target as the RISC-directing siRNA. In cleavage assays, reconstituted AGO/RISC programmed with siR gf698 catalyze the hydrolysis of the GFP mRNA into two readily distinguishable cleavage products [30,31] (Figure 1B). SiR gf698 thereby induces highly effective cleavage, i.e., fitting the definition of an *e*siRNA, in an average cleavage assay performed with plant AGO1- or AGO2/RISC, the majority of the originally added amount of target RNA is hydrolyzed (Figure 1B). To prove that DNA-directed cleavage of the GFP mRNA occurred, we performed cleavage assays, which applied the following synthetic nucleic acid guides: the individual siR gf698 guide strand; the siR gf698 duplex; ASO gf698, whose sequence corresponds to that of the siR gf698 guide strand; a double-stranded DNA oligonucleotide, ASO gf698 duplex, whose sequence as well as double-strand conformation (including 2 nt 3′-overhangs) corresponds to that of the siR gf698 duplex. On the one hand, we performed these assays in the usual manner, namely with reconstituted AGO1/RISC, where the AGO1 was derived from the plant *N. benthamiana* [20,32,33] (Figure 1A, left panel). On the other hand, we performed the assays in the absence of the in vitro-translated (additionally expressed) AGO1 (Figure 1A, right panel).

In previous, similarly performed cleavage assays in BYL, it had become clear that siRNA-mediated slicing is only observed in the case of additionally expressed AGO proteins: in other words, activities of BYL-endogenous AGO/RISC are undetectable under the applied conditions [30,31]. Accordingly, in the absence of heterologously expressed AGO, no cleavages were observed with RNA guides (Figure 1B, lanes 11–14). In contrast, in the reactions containing heterologously expressed AGO1, we observed cleavage of the target RNA in the presence of the siR gf698 duplex (Figure 1B, lanes 4 and 5). Consistent with the fact that AGO/RISC activity with a siRNA guide strand is usually only measurable after prior binding of an siRNA duplex [31], no cleavage was detectable when the reaction was performed with the siR gf698 guide strand only (Figure 1B, lanes 2 and 3). However, regardless of the presence of AGO, we observed apparent cleavages of the target RNA in the presence of the single-stranded as well as the double-stranded DNAs, albeit with lower efficiency in the latter case (Figure 1B, lanes 6–9; 15–18). Compared with the AGO1/RISC-mediated cleavage of the GFP mRNA, the DNA (ASO gf698)-directed cleavage assays yielded comparable 5′ cleavage products, while the 3′ cleavage products were either undetectable or smaller, or they appeared in a more indistinct form in the analytical gel system (see Figure 1B and subsequent figures).

These data already suggested that AGO proteins are not involved in the observed DNA-directed cleavage activity in BYL. This idea was confirmed in further cleavage assays in which we used single-stranded ASO of different lengths and with different 5′-terminal nucleotides. The data obtained here (Figure S1) showed that the detected DNA-directed cleavages of the target RNA did not depend on the type of 5′-terminus of the ASO used, nor on its length (at least in the range of the tested 19–25 nt). In addition, it became clear that pre-hybridization of the mRNA target with the ASO, i.e., effective formation of a DNA:RNA heteroduplex, significantly increased cleavage activity (Figure S1).

**Figure 2:**
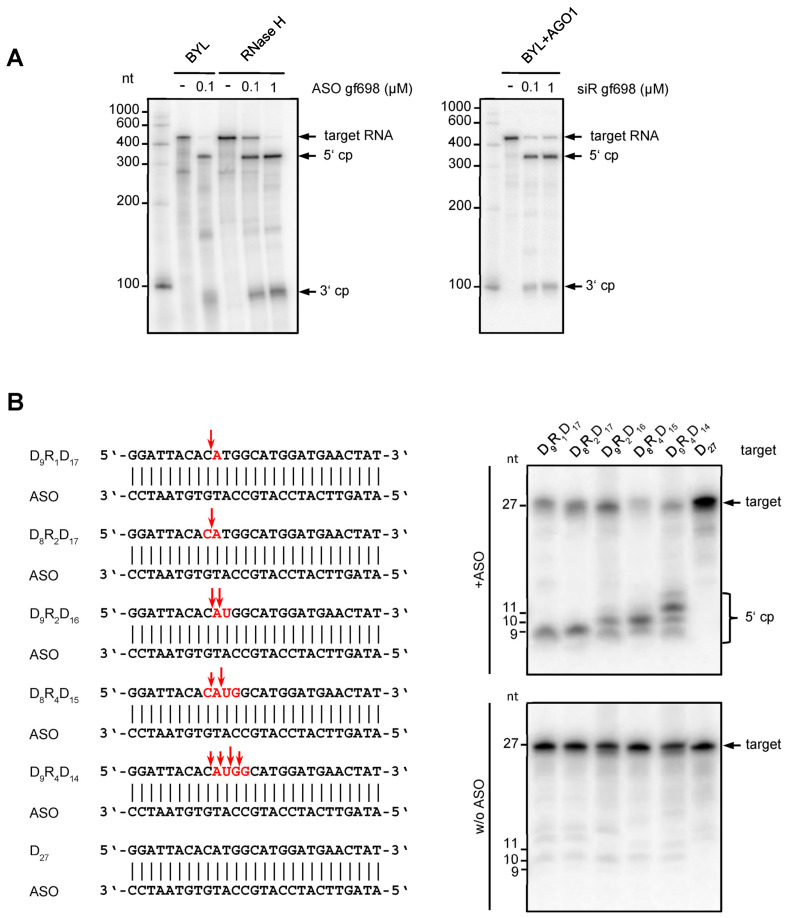
Characteristics of the DNA-directed hydrolysis activity in *Nicotiana tabacum* BYL. (**A**) The cleavage pattern of ASO-directed hydrolysis of target RNA in BYL is similar to the cleavage pattern generated by *E. coli* RNase H. ASO gf698, and the corresponding target RNA was hybridized and tested in cleavage assays in BYL, or, under analogous buffer conditions, with purified RNase H protein from *E. coli* (New England Biolabs, Frankfurt, Germany). For comparison, siRNA (siR gf698)-directed cleavage of the same target RNA was performed with reconstituted AGO1/RISC. The analyses were performed in the same way as described in Figure 1; cleavage products (cp) are indicated. (**B**) DNA-directed cleavage activity in BYL exhibits features of both RNase H1 and RNase H2. Different 27 nt DNA oligonucleotides containing one, two, or four ribonucleotides (highlighted in red) were used as targets for cleavage mediated by a complementary ASO (**left**). The design of the heteroduplexes was based on the report of Eder et al. [34]; sequences were adapted to hybridize with ASO gf698 (see Figure 1 and Section 4). The assay was performed as previously described (**upper** panel); as controls, all target nucleic acids were incubated in BYL in the absence of ASO (**lower** panel). Red arrows indicate the putative cleavage sites inferred from denaturing PAGE of total RNA isolated from the samples and subsequent autoradiography (**right**).

The observation that DNA-directed slicing of a target RNA was most effective when performed with a single-stranded ASO or with a preformed DNA:RNA heteroduplex supports the conclusion that *N. tabacum* BYL contains RNase H activities. As outlined, RNases H recognize DNA:RNA heteroduplexes and endonucleolytically hydrolyze them in the RNA component [11,12]. To test this assumption, we compared the ASO gf698-directed cleavage of GFP mRNA in BYL with the hydrolysis pattern of an ASO gf698:GFP mRNA heteroduplex using commercial, recombinant *E. coli* RNase H. Interestingly, both reactions yielded essentially the same cleavage pattern (Figure 2A). Compared again with AGO1/RISC-mediated cleavage of GFP mRNA with the siR gf698 duplex, the findings of this experiment were congruent with the previous data (Figure 1), showing that the 5′ cleavage products generated by RNA- and DNA-directed cleavage were of similar size. Conversely, the 3′ product(s) of DNA-directed cleavage appeared less defined and generally smaller than the products generated by AGO/RISC-catalyzed RNA-directed cleavage (Figure 1B and Figure 2A). Cloning of the cDNAs of the cleavage products revealed that they differed slightly in size and thus originated from different hydrolysis sites. Furthermore, identification of the 3′ end of the 5′ cleavage fragment corroborated the idea that this was generated by RNase H activity (Figure S2; further explored in experiments shown in Figure 2B).

We next wanted to understand which type(s) of RNase H was responsible for the observed activities in BYL. Two main RNase H activities, H1 and H2, were described in plants [35,36], which differ essentially in their capability to catalyze the hydrolysis of substrates composed of ribonucleotides and deoxyribonucleotides. For an endonuclease activity on a DNA:RNA heteroduplex substrate, RNase H1 requires the presence of at least four consecutive ribonucleotides. On the other hand, RNase H2 hydrolyzes heteroduplex substrates containing less than four ribonucleotides; i.e., only a single ribonucleotide in a duplex DNA is sufficient for RNase H2-mediated cleavage [12,15,37,38]. To assay BYL for both activities, we performed cleavage assays applying different types of artificial 27 nt heteroduplexes. These consisted of a DNA strand interspaced with different numbers of consecutive ribonucleotides and the corresponding fully complementary ASO (Figure 2B). Interestingly, heteroduplex substrates containing less than four ribonucleotides were clearly hydrolyzed in the assays, which was taken as a clear indication of RNase H2 activity (Figure 2B) [35,36,37,38]. However, for the substrates that contained four consecutive ribonucleotides and where RNase H2 was expected to cleave between the first and second and between the third and fourth ribonucleotides, leaving one ribonucleotide attached to each DNA fragment, we also observed cleavages at additional positions (Figure 2B). As these cleavages occurred preferentially between the second and third ribonucleotide, we interpreted these observations as an indication of RNase H1-mediated hydrolysis [35,36,37,38]. Taken together, our data revealed that the *N. tabacum*-derived BYL exhibits DNA-directed RNA hydrolysis activities, which involves both RNase H2 and RNase H1. The involvement of multiple RNase H enzymes in ASO-mediated cleavage explains the previously observed inhomogeneity of cleavage products.

### 2.2. ASOs Derived from Antiviral esiRNAs Provide Antiviral Protection

The above in vitro experiments with an applied model target RNA, an *e*siRNA, and an ASO whose sequence was derived from that of the *e*siRNA, suggested that ASO-mediated hydrolysis catalyzed by RNase H was comparably effective with siRNA-mediated hydrolysis catalyzed by AGO/RISC (Figure 1 and Figure S1). This suggests that both types of nucleic acid-directed endonucleases exhibit very homologous behavior on a target RNA despite their very different mechanisms of action. One hypothesis that might explain these similarities in the reaction pattern of AGO/RISC and RNase(s) H is that the activities of both endonucleases require base pairings of short NA with accessible sites (a-sites) in the target RNA. To further explore this hypothesis, we extended our analysis to a natural, more complex RNA target, the genome of Tomato bushy stunt virus (TBSV). TBSV, which belongs to the family *Tombusviridae*, is a positive-strand RNA virus, i.e., the genome itself is infectious; after cell entry, it acts directly as mRNA and template for RNA replication in the cytoplasm of the host cell. Because of its broad tropism and distinct pathogenesis, TBSV is particularly well-suited for studying the effects of antiviral silencing [39]. In a previous screen using the BYL system, we identified a series of *e*siRNAs, siR179, 209, 3243, and 3939 (numbering corresponds to the position of the 5′-end of the siRNA’s guide strands when hybridized to the TBSV RNA), which induce effective AGO1- or AGO2/RISC-mediated hydrolysis of the 4.8 kb TBSV genome. Most importantly, in different types of applications, i.e., Agrobacteria/amiR-mediated expression or “*rub-inoculation*”, these *e*siRNAs were shown to have significant protective effects against TBSV infections in *N. benthamiana* plants [20]. Accordingly, we decided to test ASOs whose DNA sequences corresponded to those of the guide strands of TBSV-targeting *e*siRNAs in in vitro and in vivo silencing experiments. As negative controls, we used ASOs whose sequences matched the guide strands of two TBSV siRNAs, siR3701 and 3722, which, during the screening procedure, showed neither slicing in vitro nor antiviral activity in planta [20].

**Figure 3:**
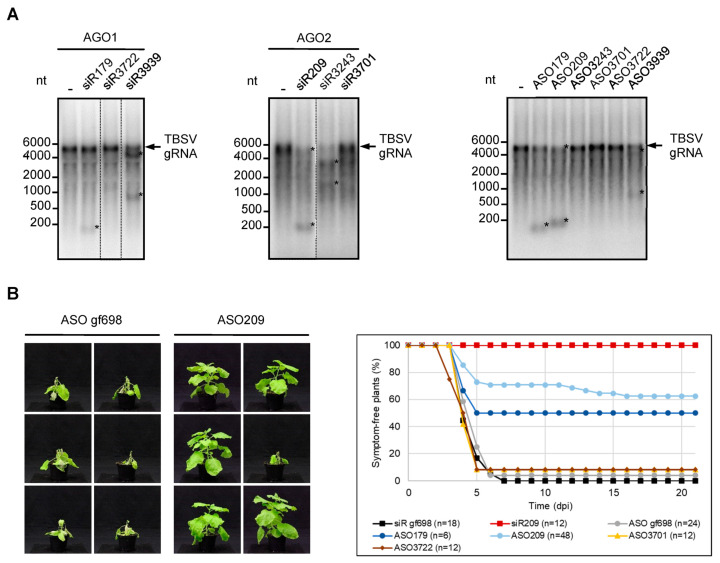
ASOs derived from *e*siRNAs targeting the TBSV genome show antiviral activity. (**A**) ASOs derived from *e*siRNAs mediate efficient hydrolysis of viral RNA in vitro. Cleavage assays with TBSV-targeting siRNAs siR179, siR3722, and siR3939 that were previously shown to function efficiently in AGO1/RISC and siRNAs siR209, siR3243, and siR3701 that were previously shown to function efficiently in AGO2/RISC [20]. *N. benthamiana* AGO1 or AGO2 were generated by translation in BYL in the presence of the associated siRNA duplexes, thus generating RISCs that were programmed with these siRNAs. Subsequently, labeled full-length TBSV genomic RNA was added and analyzed for siRNA-mediated cleavage by denaturing agarose gel and autoradiography (**left**, **middle**). ASOs corresponding in sequence to the guide (antisense) strand of the aforementioned siRNAs were used in an analogous assay designed to analyze RNase H-mediated hydrolysis of TBSV RNA (**right**). The assay was performed in the same manner as described above, except that no AGO mRNAs were added to BYL (see also Figure 1). Asterisks indicate cleavage products. (**B**) ASOs corresponding in sequence to the guide strand of TBSV-targeting *e*siRNAs protect plants against TBSV infections. *N. benthamiana* plants were co-inoculated with genomic TBSV RNA and with TBSV-targeting siRNAs or ASOs using the *rub-inoculation* procedure (see Section 4). SiR gf698 and the corresponding ASO gf698 were used as negative controls; siR209 was applied as a positive control. Additional controls involved ASO3701 and ASO3722; their sequences were derived from siRNAs directed against the TBSV genome but were nonfunctional in vitro (see **A**). The plants were monitored for 21 days post inoculation (dpi) for the appearance of typical symptoms of virus infection (representative pictures on the left). Except for ASO179, minimally, two independent infection experiments were performed for each nucleic acid.

First, we performed cleavage assays comparing the *e*siRNA/AGO- and ASO/RNase H-induced hydrolysis of full-length TBSV RNA. As expected, all of the above *e*siRNAs were active and effective when incorporated into AGO1 or AGO2/RISC (Figure 3A). Interestingly, all ASOs whose sequence matched that of the guide strands of these *e*siRNAs, with one exception (ASO3243), also induced marked hydrolysis of TBSV RNA in the BYL. In contrast, ASO3701 and ASO3722, which matched the guide strands of the two siRNAs that were earlier determined to be inactive, did not cause cleavage of TBSV RNA (Figure 3A).

In a second experiment, we tested for the potential antiviral activity of ASO in planta. Using a standard protocol, we rub-inoculated the leaves of 4–6 weeks-old *N. benthamiana* plants with in vitro transcribed TBSV genomic RNA. The rub-in mixture was supplemented with ASO gf698 as a non-specific control, ASO3701 and ASO3722 were previously found to be inactive in the DNA-directed cleavage assays (specific negative controls; see Figure 2), and ASO179 and ASO209 which, as its *e*siRNA-counterparts siR179 and siR209, induced the most effective RNA cleavage in vitro (Figure 3A). In parallel, we performed the analogous plant experiments with siR gf698 (negative control) and siR209 (positive control). After inoculation, plants were monitored for three weeks for the appearance of typical infection symptoms, ranging from crinkled younger leaves to severe systemic necrosis. As shown in Figure 3B, almost all plants that received the TBSV RNA, along with the non-specific control ASO or control siRNA, as well as those that received ASO3701 or ASO3722, developed distinct symptoms within the observation period. In contrast, the majority of TBSV-infected plants treated with ASO209 and half of the plants treated with ASO179 showed no symptoms within the three weeks examined. Application of siR209 provided the best protection, i.e., all treated plants remained symptom-free. These data are consistent with previous observations where siR209 showed the best protective effect against TBSV infection out of a number of different *e*siRNAs [20] (Figure 3B).

In summary, we can draw several important conclusions from these results. First, our data support the conclusion that regions of a target RNA identified in vitro as accessible to siRNA/RISC (a-sites) are indeed comparably accessible to ASO/RNase H. Second, our results have shown that ASOs, and thus RNase H-mediated cleavage of target RNAs may be well suited for plant protection against viruses. Third, in close analogy to what was previously found for siRNAs, a direct correlation was observed between the cleavage efficiency exhibited by an ASO on a complex viral target RNA in vitro and its protective effect in planta. In other words, ASOs that were effective in vitro were also effective in planta and thus proved to be useful antiviral agents. Conversely, ASOs that were ineffective in vitro were also ineffective in planta. Thus, both RNA-guided AGO/RISC-mediated as well as DNA-guided RNase H-mediated silencing of a target RNA in vitro is a reliable indicator of the protective effect of the respective guide nucleic acid in planta. Following the previous terminology, we will refer to the effective ASOs as *e*ASOs in the following.

### 2.3. Chemical Modification Increases the Antiviral Effect of eASO 

The effect of ASOs, e.g., on post-transcriptional gene expression in mammals, can be significantly enhanced by chemical modifications of the oligonucleotide affecting either individual nucleotide bases or the phosphate backbone [10,11]. Accordingly, the next experiments should investigate whether the antiviral silencing capacity of an *e*ASO can be affected by common chemical modifications. For this purpose, we applied ASO209 variants containing three types of modification, namely a modification at the 2′ oxygen of the ribose (2′-O-methoxyethyl (MOE4)), a “locked nucleic acid (LNA2)” modification with a methylene bridge bond between the 2′ oxygen and the 4′ carbon of the ribose, and phosphate backbone modifications where we replaced the two terminal phosphodiester bonds (PS2) or all (PS20) phosphodiester bonds of ASO209 by phosphorothioates. As expected from similar data in mammalian cells and cell extracts [40], all except LNA2 increased the stability of the *e*ASO in BYL. This was most pronounced for PS20 (Figure S3).

**Figure 4:**
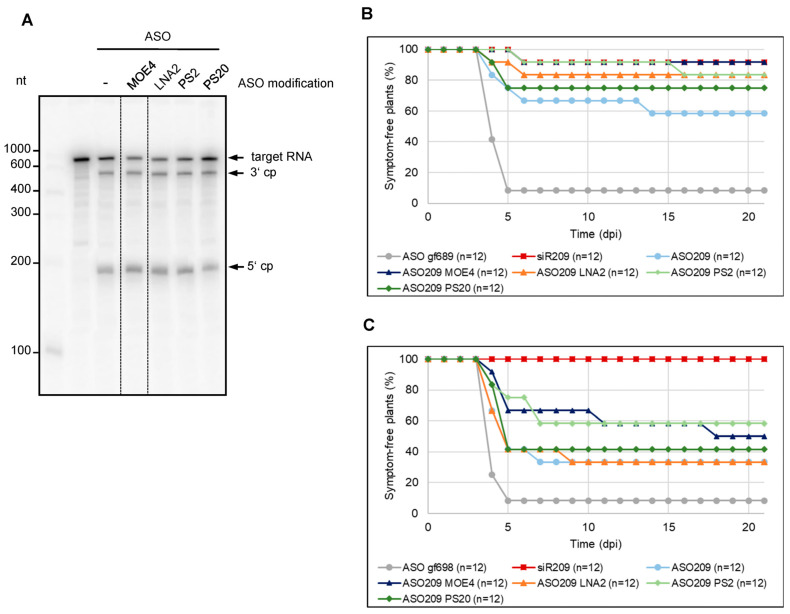
Chemical modifications increase the antiviral activity of ASO. (**A**) Cleavage assays with chemically modified ASOs. Variants of ASO209 with 2′-O-methoxyethyl modifications at the four terminal nucleotides (two at the 5′ end, as well as at the 3′ end, referred to here as MOE4), locked nucleic acid modifications at the two terminal nucleotides (LNA2), phosphorothioate modification at the two terminal phosphodiester bonds (PS2), or at all phosphodiester bonds (PS20) were used to analyze the RNase H-mediated hydrolysis of the 5′ part of TBSV genomic RNA containing the ASO binding site. The ASO and the ^32^P-labeled target RNA (encompassing the 5′-terminal 740 nucleotides of the TBSV genome) were combined before being added to BYL. Cleavage was analyzed by denaturing PAGE. Cleavage products (cp) are indicated by arrows. (**B**) Chemical modifications improve the *e*ASO-mediated protection of plants against a TBSV infection. *N. benthamiana* plants were co-inoculated with TBSV genomic RNA and with ASO209 or ASO209 variants by rubbing the nucleic acids onto the leaves. ASO gf698, targeting GFP mRNA, was used as a negative control, and siR209 was applied as a positive control. The plants were monitored for 21 days post-inoculation (dpi) for the appearance of typical symptoms. Two independent infection experiments were performed. (**C**) Sequential inoculation of ASOs and viral RNA also protects plants from viral infection. *N. benthamiana* plants were first inoculated with ASO209, chemically modified variants of ASO209 or the aforementioned controls. Three minutes later, TBSV genomic RNA was rubbed onto the same leaves. The plants were monitored for 21 days post-inoculation (dpi) for the appearance of disease symptoms. Two independent infection experiments were performed.

When we first tested these ASO variants for their capability to induce RNase H-mediated cleavage of target TBSV RNAs, we found all of them to be effective. This was demonstrated with a TBSV RNA corresponding to the 5′-terminal portion of the viral genome containing the complementary ASO hybridization site (Figure 4A) as well as with the full-length genomic viral RNA (Figure S4). Interestingly, this was observed regardless of whether ASOs and TBSV RNA were pre-incubated simultaneously in BYL or whether ASO was added to BYL first and TBSV RNA was added afterwards (Figure S4). We took this as an indication that DNA:RNA heteroduplex formation can occur under very different conditions.

When we next examined the protective effect of the modified ASOs against TBSV infection in planta using essentially the same inoculation protocol as above, we interestingly found that all modifications tested increased the protective effect of ASO209. While ca. 60% of the plants remained symptom-free with the unmodified ASO, about 80–90% did so with the MOE4-, PS2-, and LNA2-modified ASO209. With PS20 modification, ca. 75% of the plants remained symptom-free. Thus, the ASOs modified in this way had a protective effect comparable to siR209, which was again used as a control (Figure 4B).

In each of the infection experiments performed previously, treatment with ASOs was carried out in such a way that the infectious viral RNA was co-inoculated with the DNA (Figure 3B and Figure 4B). This procedure implied that the inoculation occurred predominantly with a preformed DNA:RNA heteroduplex. In view of our previous observation that ASO:target RNA heteroduplex formation can occur under very different conditions, the final question of this study was whether binding of an identified *e*ASO to an infectious viral RNA can also occur separately in time. This would clarify, on the one hand, whether *e*ASO can associate with complex viral RNA molecules under conditions other than in vitro. On the other hand, clarification of this question was important with regard to the development of further strategies for possible treatment procedures with *e*ASO, not least for the treatment of plants. To this end, we first treated the leaves of *N. benthamiana* plants in the usual manner with the chemically modified ASO209 variants. Following a procedure similar to that previously described for the treatment of plants with dsRNA [41], we then inoculated the same site with the genomic TBSV RNA at a later time point. Conducting these inoculation experiments over the usual time intervals (see also Figure 3B and Figure 4B) showed that protection with ASO also worked under these conditions: although the total number of plants protected was lower than in the previous experiment, we still observed an adequate level of protection of about 60% of the plants, for example, with ASO209 PS2 and ASO209 MOE4 (Figure 4C).

In summary, our data show that *e*ASO identified in the manner described, namely via a-sites in target RNAs that appear to be generally accessible to endonuclease/NA complexes, can be extremely useful tools for approaches aimed at inactivating complex RNA molecules such as the RNA genomes of plant viruses.

## 3. Discussion

Target RNAs of siRNAs and ASO, such as mRNAs or viral genomic RNAs, although usually referred to as single-stranded, are predominantly not, but rather form higher-order secondary or tertiary structures [42]. In cellular mRNAs, and especially in viral genomes, these structures have essential biological functions; for example, they serve as initiation and or regulatory regions for translation and/or RNA replication [43]. In particular, viruses that co-evolved with the cellular RNA-silencing mechanism in cells, as is the case with plant viruses, can be assumed to have a “target RNA folding” that severely limits effective RNA silencing. In addition, it can be assumed that all types of RNA-binding proteins significantly affect RNA structure and a-site availability. This explains the previous observations by others and by ourselves [20,44], which showed that DCL activity on viral genomic RNAs in plant cells, or cell extracts, generates a large variety of siRNAs, but only a small fraction of them are effective. The problem of complex folding of target RNAs also affects all kinds of design strategies aimed at identifying effective siRNAs or ASOs. Insufficient attention to this aspect, especially due to unreliable prediction methods, explains the failure of many siRNA and ASO candidates, and the efficacy of nucleic acid drugs already in use could potentially be much better.

As explained earlier, we have recently developed an experimental method that allows us to identify from a large pool of siRNAs reliably *e*siRNAs that are very effective in RNAi. Proof-of-concept was first obtained for the TBSV RNA genome [20] and recently confirmed for other viral and cellular mRNA targets. With TBSV, we could directly match our identified *e*siRNAs to the genomic RNA structure, which was experimentally determined by Wu et al. [43]. However, the binding sites of several identified *e*siRNAs (e.g., siR179 and siR209) are located in partial RNA double strands and, thus, in regions of this structure that are not obviously accessible (Figure S5). This indicates that the experimental structure of the TBSV genome appears to form in only a few cases or not at all under physiological conditions. However, the opposite appears to be true in BYL, where complex target RNAs appear to adopt the structures they do under native conditions (i.e., in the cell): indeed, there is currently no other explanation for the observed striking correlation between the in vitro and in vivo data on the antiviral efficacy of *e*siRNAs. Clearly, these findings, so far purely empirical, require detailed structural analyses of various target RNAs in BYL in future studies to reach further conclusions here.

Here, we show that this method, now referred to as the “*e*NA screen”, can also be applied to the design of ASOs capable of effectively triggering RNase H-mediated RNA silencing of target RNAs. As demonstrated, this is possible by very simple derivation of the ASO sequence from the sequence of previously identified *e*siRNAs guide strands. This was made possible by the interesting fact that BYL, in addition to functional translational machinery and active DCLs, also contains active RNase H2- and RNase H1-related RNases H (Figure 2). Application of the *e*ASOs determined in this way in antiviral plant protection experiments again showed the close correlation between the observed in vitro cleavage efficiency and in vivo protective efficacy already described for *e*siRNAs. However, we also found that some *e*siRNA-derived ASOs were not active, again fitting the scenario that the AGO/RISC and RNase H endonucleases are active in silencing via different mechanisms. Nevertheless, *e*NA screening proved convincingly useful in identifying siRNAs and ASOs, which are effective in silencing complex RNA molecules (Figure 3). We take the fact that both NAs can be identified in the same approach as a clear indication that both silencing mechanisms based on small NAs and a guided endonuclease follow fundamentally similar rules—the recognition of accessible sites in the complex RNA molecules. These “a-sites” can apparently be effectively determined by the applied screening procedure. Thus, in summary, an “a-site-based approach” became available for the future design of siRNAs and ASO that are effective in silencing. We suggest this approach to also be applicable to other complex-folded target RNAs, such as genomes of animal and human viruses, as well as cellular mRNA addressing all types of RNA- and DNA-antisense-based knockdown processes. It will be very interesting to understand whether the “a-site paradigm” is also applicable to other NA-guided endonucleases, such as Cas proteins [45].

In this study, we also show the value of chemical modifications in the context of the *e*NA screen by testing “different generations” of modifications known to affect very different properties of ASO, such as stability (see Figure S4), hybridization efficiency with the target, and cell-to-cell transferability [10,11]. In our experimental systems, it was very clear that ASO modifications led to a significant improvement in antiviral activity both in vitro and in vivo. Indeed, the activity of an *e*ASO, which was lower compared with the corresponding *e*siRNA (Figure 3), could thus be raised to almost the same level (Figure 4). The same series of experiments (Figure 4C) showed that initial heteroduplex formation outside of the plant is not required for *e*ASO applications, which is of immense importance for further applications. Further systematic studies are required to achieve maximal activities of *e*ASO/RNase H complexes at accessible sites of complex target RNAs. Crucial at this point, however, was the breakthrough possibility of identifying *e*ASOs and the observation that their activity can be further enhanced by chemical modification.

The *e*ASOs identified here have been successfully tested in antiviral approaches in plants. In fact, the application of ASOs in plants is only slowly gaining interest. Mostly used to study gene function, the recent development of methods to introduce ASOs into plants, e.g., by infiltration, has opened up further applications ([46,47,48,49]; reviewed in [50,51]). Apparently, both types of RNases H (H1 and H2) are present in planta [35], which was confirmed here with the BYL system (Figure 2). Accordingly, ASOs can be adapted in a manner similar to what was possible with human RNase H variants [11], e.g., by designing gapmers to meet the requirements of the plant enzymes much better than was the case with the approaches shown here. Thus, there is still considerable room for optimization here as well. This aspect must also be seen in light of the fact that the RNase H proteins and the AGO proteins involved in RNA silencing, although both have an RNase H motif and are active in the cytoplasm, act enzymatically and biologically quite differently, resulting in different pharmacological properties and behaviors. For example, human RNase H1 is remarkably specific for RNA/DNA-like duplexes and for specific sequences: the enzyme uses all the information contained in an ASO and is sensitive to the sequence and helical structure of the heteroduplex at the catalytic site [52]. These properties imply a very low propensity for off-target cleavages. In contrast, AGO proteins are more promiscuously designed; they mainly use the seven-nucleotide seed region to discriminate between target and non-target RNAs. ASO-mediated RNA silencing is, therefore, much less susceptible to off-target effects [53].

In addition to better target specificity, ASOs are easier and cheaper to synthesize than siRNAs or dsRNAs, and in the human system, ASOs have been shown to have low immunoreactivity, which is also of significant importance for potential applications in edible plants. There are no differences between the use of ASO and RNA molecules with regard to the safety aspect; to our knowledge, there are no findings in the numerous human clinical studies that prove, for example, genomic integration events attributable to the use of ASO. Since 1998, several ASOs have been approved for clinical use by the FDA, providing strong evidence of the safety of ASO drugs [13]. Thus, ASOs could be a relevant alternative to topical applications of nucleic acid-based drugs in plants, targeting viruses but also other plant pathogens such as insects. In the latter case, there is even hopeful evidence that ASOs can actually be used as pesticides [54].

## 4. Materials and Methods

### 4.1. Cell Culture and Preparation of BYL 

*Nicotiana tabacum* BY2 cells were cultured at 23 °C in Murashige-Skoog liquid medium (Duchefa, Haarlem, The Netherlands). Cytoplasmic extracts (BYL) were prepared from evacuolated cells as described [28,29].

### 4.2. In Vitro Transcription 

*N. benthamiana AGO* mRNAs were synthesized in the presence of monomethylated cap analog m^7^GP_3_G (Jena Biosciences, Jena, Germany) from SwaI-linearized plasmid constructs [20,30] using SP6 RNA polymerase (Thermo Fisher Scientific, Waltham, MA, USA). Transcripts encoding the firefly luciferase mRNA were generated by SP6 RNA polymerase from the XhoI-linearized plasmid pSP-luc(+) (Promega, Madison, WI, USA). Transcription reactions and subsequent treatment of the transcripts were performed by using standard procedures. TBSV genomic RNA (T100) was synthesized by T7 RNA polymerase from a SmaI-linearized plasmid [29,55]. The 5′ terminal fragment of TBSV RNA and the GFP mRNA fragment that were used as target RNAs in cleavage assays were produced by T7 RNA polymerase from PCR products, whereby the T7 promoter sequence was included in the forward PCR primer (see Supplementary Tables S1 and S2 for oligonucleotide sequences). Labeling of RNAs was performed by in vitro transcription in the presence of 0.2 µCi/µL [α-^32^P]-CTP (3000 Ci/mmol).

### 4.3. Oligonucleotides and siRNAs

Unmodified and MOE-modified DNA oligonucleotides were purchased from Microsynth AG (Balgach, Switzerland). DNA/RNA hybrids were synthesized by Microsynth AG or Biomers (Ulm, Germany). LNA- and PTO-modified DNA oligonucleotides were purchased from Sigma Aldrich (Taufkirchen, Germany). The siRNA siR gf698 targeting GFP mRNA and siRNA siR209 targeting TBSV were described earlier [20,30,31]. RNA oligonucleotides to generate the siRNAs were purchased from Biomers. To produce siRNA duplexes, both strands were mixed in siRNA annealing buffer (30 mM Hepes/KOH, pH 7.4, 100 mM KOAc, 2 mM MgOAc) and incubated for 1 min at 90 °C and for 60 min at 37 °C. Double-stranded DNA oligonucleotides were produced by heating both strands in DNA annealing buffer (10 mM Tris/HCl pH 7.5; 100 mM NaCl; 1 mM EDTA), followed by cooling down to 25 °C within ca. 30 min. Labeling of oligonucleotides was performed using T4 polynucleotide kinase (Thermo Fisher Scientific) according to the manufacturer’s protocol and 0.5 µCi/µL [γ-^32^P]-ATP (3000 Ci/mmol). All DNA and RNA oligonucleotide sequences are listed in Supplementary Tables S1 and S2.

### 4.4. In Vitro Cleavage Assays

Cleavage assays were performed in a 20 µL reaction containing 50% (*v*/*v*) BYL using the previously described conditions [30,31,32,33]. AGO/RISC-mediated cleavage of target RNAs was performed by translation of 0.5 pmol *AGO* mRNA (encoding AGO1 or AGO2) in the presence of 0.1 or 1.0 µM siRNA guide (antisense) strand or siRNA duplex. After 2.5 h at 25 °C, 2 µg of firefly luciferase (competitor) mRNA and the ^32^P-labeled target RNA (50 fmol) were added, and the cleavage reaction was performed for 15 min. RNase H-mediated cleavage of target RNAs was achieved as described above but without the addition of *AGO* mRNA and in the presence of 0.1 or 1.0 µM antisense DNA oligonucleotide or the respective duplex (see also Figure 1). Alternatively, target RNA and DNA oligonucleotide were mixed, incubated at room temperature for 5 min and added to the BYL reaction mixture for 15 min at 25 °C. In each case, total RNA was isolated by treating the reaction with 20 µg proteinase K in the presence of 0.5% SDS for 30 min at 37 °C, followed by chloroform extraction and ethanol precipitation. RNAs from assays with full-length TBSV RNA as targets were separated on 1.5% denaturing agarose gels; all other samples were separated on 6–15% TBE polyacrylamide gels containing 8 M urea. ^32^P-labeled target RNAs and cleavage products were visualized by phosphor-imaging (Typhoon Trio+, GE Healthcare, Chalfont St Giles, UK).

The cleavage assay with purified *E. coli* RNase H (New England Biolabs, Frankfurt, Germany) was performed with 0.1 or 1.0 µM of antisense DNA oligonucleotide pre-mixed with target RNA. This mixture was incubated in a 20 µL reaction with 2.5 units of enzyme and the supplied reaction buffer for 20 min at 37 °C. 10 µL of the reaction mixture was directly analyzed on a denaturing gel as described above.

### 4.5. 3′-Rapid Amplification of cDNA Ends (3′-RACE)

3′-RACE was used to identify the 3′ end of the 5′ cleavage fragment of GFP mRNA. The cleavage products were separated on a 6% (*w*/*v*) denaturing TBE polyacrylamide gel, and the larger fragment was eluted from the gel. The purified RNA was ligated to the Universal miRNA cloning linker (New England Biolabs) using T4 RNA ligase (Promega). The ligation product was reverse-transcribed using AMV RT (Promega) and an oligonucleotide primer complementary to the linker sequence. The cDNA was amplified by PCR using a forward primer located in the known 5′ region of the fragment and cloned into the pGEM-T Easy vector (Promega). The resulting plasmids were sequenced.

### 4.6. Stability of ASOs and siRNAs in BYL

10 nM of 5′ labeled DNA oligonucleotides or siRNA duplex were incubated for the indicated times in 50% (*v*/*v*) BYL using the conditions previously applied in cleavage assays [33]. Total RNA was isolated from the reactions and analyzed as described above using 15% (*w*/*v*) denaturing TBE polyacrylamide gels.

### 4.7. Plant Treatment, TBSV Challenge, and Analysis of Infection Assays

Mechanical co-inoculation of siRNAs or ASOs and TBSV genomic RNA was performed with 4-week-old *N. benthamiana* plants. Prior to the application of the RNAs, the upper surface of the third and fourth leaf was dusted with carborundum powder. 10 µL solution containing TBSV RNA (5 or 10 ng) and siRNA (150 pmol) or DNA oligonucleotide (900 pmol) were mixed with an equal volume of inoculation buffer (30 mM K_2_HPO_4_, pH 9.2, 50 mM glycine) and 5 µL were rubbed onto each leaf half. Alternatively, the indicated amounts of siRNA or ASO were inoculated first, and TBSV RNA was applied 3 min later to the same leaves. The treated leaves were rinsed with water, and the plants were grown in a chamber (CLF Plant Climatics, Wertingen, Germany) for 14 h at 23 °C, 90–100 µmolm^−2^ s^−1^ light (at shelf level) and for 10 h at 21 °C in the dark. The plants were monitored daily for three weeks for the development of symptoms. The analysis of the infection assays was based on the simple distinction of symptomatic/symptom-free. In the *N. benthamiana*/TBSV system used, this is very easy to achieve by detecting obvious symptoms such as systemic necrosis. RNA accumulation was not monitored, as we knew from previous work that in plants that are still symptom-free 3–5 weeks after inoculation with TBSV, viral RNA is usually not detectable in the upper, non-inoculated leaves [20].

## Data Availability

Data are contained within the article and supplementary materials.

## Supplementary Materials

The supporting information can be downloaded at: https://www.mdpi.com/article/10.3390/ijms242417153/s1.
