# Supplementary material for: Effective Antiviral Application of Antisense in Plants by Exploiting Accessible Sites in the Target RNA
Source: Int J Mol Sci. 2023 Dec 5;24(24):17153. doi: 10.3390/ijms242417153 (PMC10743417; doi:10.3390/ijms242417153)
Supplement: Supplementary file 1 [file ijms-24-17153-s001.zip › ijms-2696641-supplementary.pdf]

Figure S1

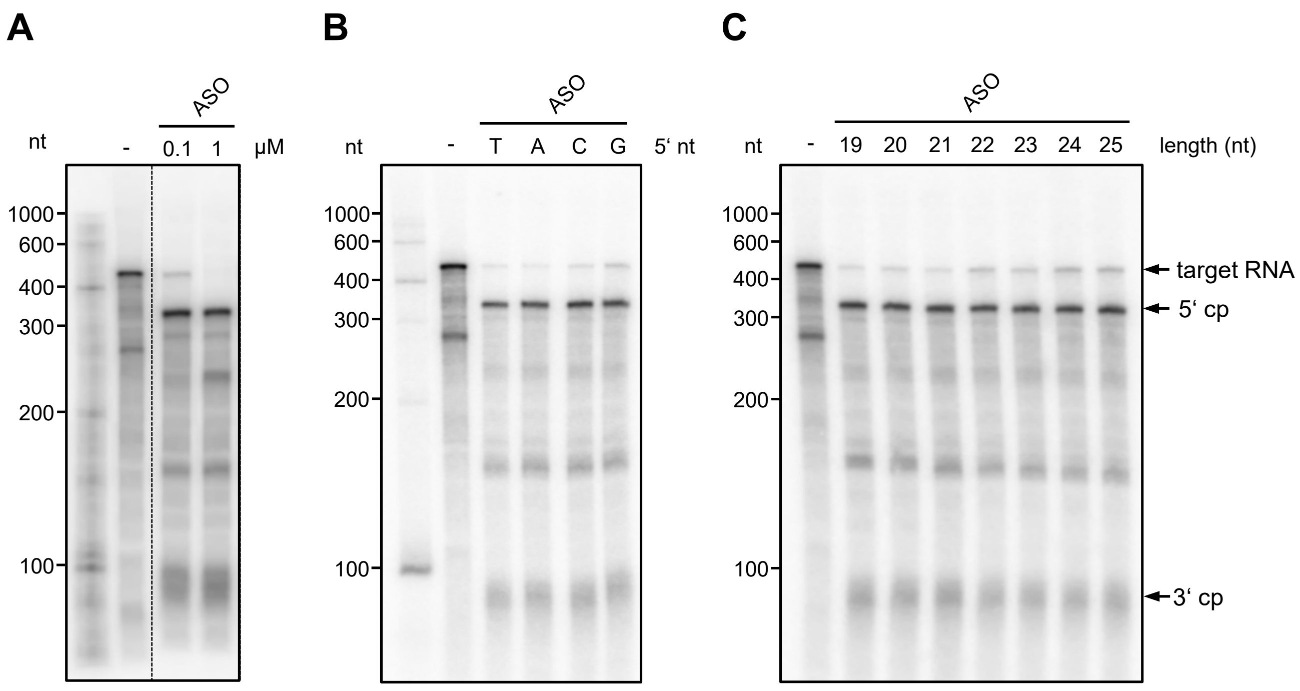

Figure S1. DNA-directed cleavage activity in BYL is enhanced by pre-incubation of ASO with target RNA and does not depend on the length or the 5'-terminal nucleotide of the ASO. (A) ASO gf698-mediated cleavage pf GFP target RNA. Unlike the cleavage assays shown in **Fig. 1**, where the ASO used was added directly to the plant extract, here the indicated amounts of ASO gf698 were hybridized with the labeled target RNA, and the preformed DNA:RNA hybrid was added to the BYL. Cleavage of the target RNA was analyzed as in **Fig. 1**. Since the cleavage efficiency of the target RNA observed here is significantly higher than in **Fig. 1**, it can be concluded that the preformed DNA:RNA hybrid significantly increases the efficiency of the reaction **(B)** Cleavage assays performed with 0.1 µM of ASO gf698 variants with different 5' nucleotides. **(C)** Cleavage assays performed with 0.1 µM of ASO gf698 variants of different lengths. Cleavage products (cp) are indicated.

Figure S2

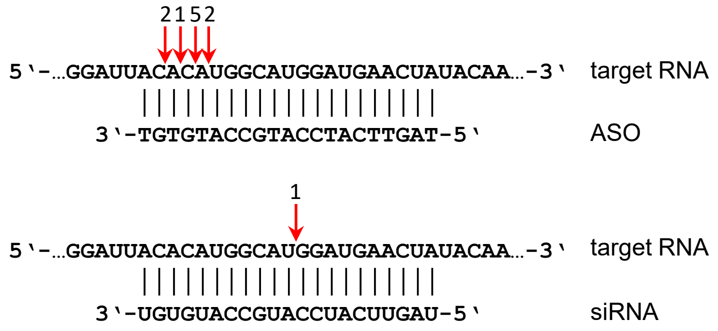

Figure S2. Mapping of cleavage sites generated by the potential RNase H activity in BYL. ASO gf698-mediated hydrolysis of a fragment of the mRNA of Green fluorescent protein (GFP) was performed in BYL as describe above, siRNA gf698-mediated cleavage was carried out in the presence of additional, *in vitro* translated AGO1 protein. 5' cleavage products were isolated from the reactions and analyzed by cloning the corresponding cDNA via 3' RACE and subsequent sequencing of the resulting plasmids. Arrows show putative cleavage sites in the target RNA, digits above the arrows indicate the number of plasmid sequences that revealed a cleavage event at the respective position.

Figure S3

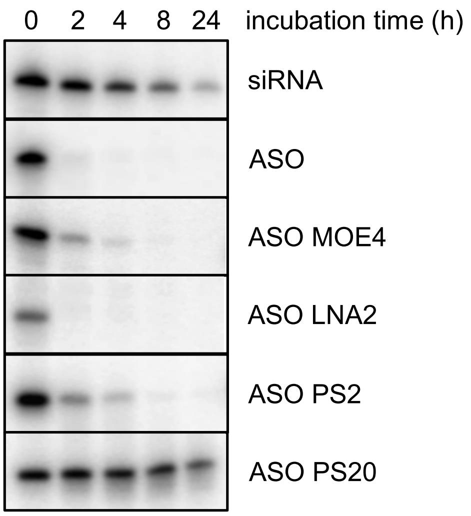

Figure S3. Effects of nucleotide modifications on ASO stability in BYL. Defined quantities of ^{32}P-labeled siRNA 209 duplex, ASO209 and ASO209 variants with different nucleotide modifications (see **Fig. 4**) were incubated in BYL for 0, 2, 4, 8 and 24 h and their stability checked by denaturing PAGE and autoradiography.

Figure S4

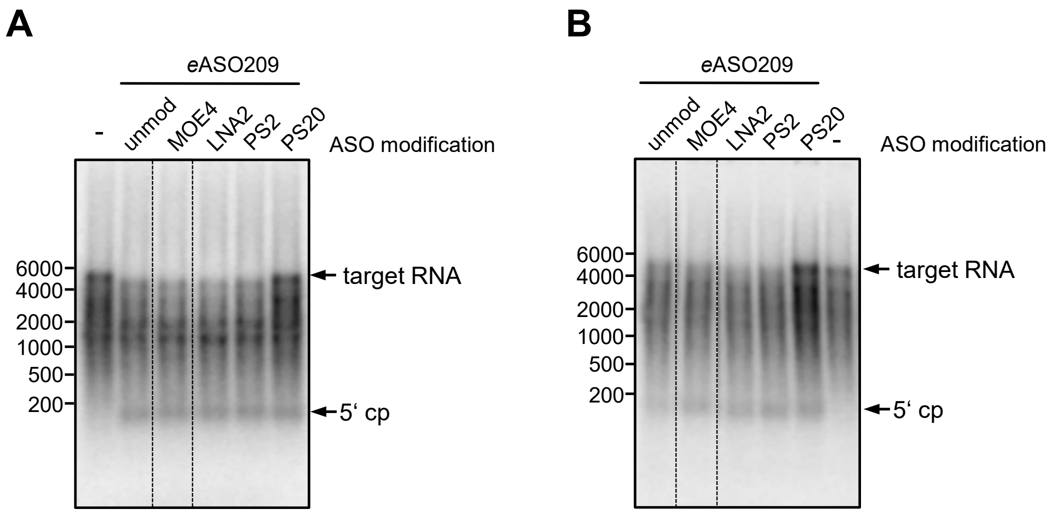

Figure S4. ASO modifications do not impair the ASO-mediated hydrolysis of a target RNA. Cleavage assays with chemically modified ASOs. Variants of ASO209 with 2'-O-methoxyethyl modifications at the four terminal nucleotides (two at the 5' as well as at the 3' end, referred to here as MOE4), locked nucleic acid modifications at the two terminal nucleotides (LNA2), phosphorothioate modification at the two terminal phosphodiester bonds (PS2) or at all phosphodiester bonds (PS20) were used to analyze the RNase H-mediated hydrolysis of full-length TBSV genomic RNA in comparison with the unmodified (unmod) ASO. **(A)** ASOs and ^{32}P-labeled target RNA were combined before adding both to BYL. **(B)** ASOs were incubated in BYL for 2,5 h before adding the ^{32}P-labeled target RNA. Cleavage was analyzed by denaturing PAGE of total RNA and subsequent autoradiography. 5' cleavage products (cp) are indicated by arrows.

Figure S5

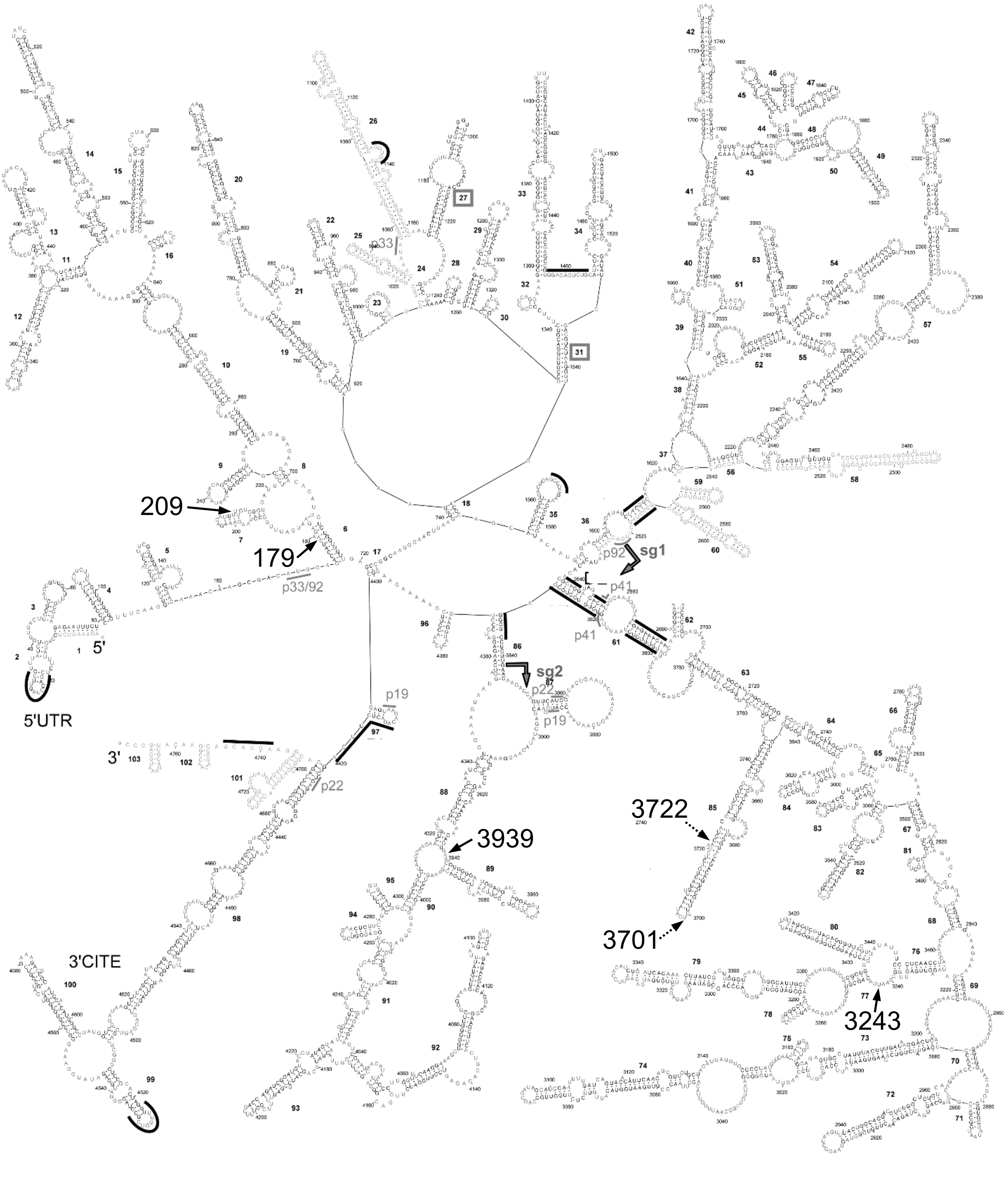

Figure S5. Experimentally determined structure of a TBSV genomic RNA [43]: binding sites of *e*siRNAs. The binding sites of *e*siRNAs siR179, siR209, siR3243 and siR3939 are indicated by solid arrows. Putative binding sites of inactive siRNAs siR3702 and 3722 are indicated by dashed arrows.

Supplementary Table S1. DNA oligonucleotides and DNA/RNA hybrid oligonucleotides used in this study

oligonucleotide	sequence[a]	description
ASO gf698	TAGTTCATCCATGCCATGTGT	ASO targeting the mRNA of Green Fluorescent Protein (GFP)
ASO gf698 (s)	ACATGGCATGGATGAACTATA	sense-strand to generate ASO gf698 duplex
ASO gf698 (5'A)	AAGTTCATCCATGCCATGTGT	variant of ASO gf698 with 5'-terminal A
ASO gf698 (5'C)	CAGTTCATCCATGCCATGTGT	variant of ASO gf698 with 5'-terminal C
ASO gf698 (5'G)	GAGTTCATCCATGCCATGTGT	variant of ASO gf698 with 5'-terminal G
ASO gf698 (19 nt)	TAGTTCATCCATGCCATGT	19 nt variant of ASO gf698
ASO gf698 (20 nt)	TAGTTCATCCATGCCATGTG	20 nt variant of ASO gf698
ASO gf698 (22 nt)	TAGTTCATCCATGCCATGTGTA	22 nt variant of ASO gf698
ASO gf698 (23 nt)	TAGTTCATCCATGCCATGTGTAA	23 nt variant of ASO gf698
ASO gf698 (24 nt)	TAGTTCATCCATGCCATGTGTAAT	24 nt variant of ASO gf698
ASO gf698 (25 nt)	TAGTTCATCCATGCCATGTGTAATC	25 nt variant of ASO gf698
target D_{27}	GGATTACACATGGCATGGATGAACTAT	27 nt DNA substrate for RNase H assay
target $D_9R_1D_{17}$	GGATTACACATGGCATGGATGAACTAT	DNA/RNA hybrid substrate for RNase H assay
target $D_8R_2D_{17}$	GGATTACACATGGCATGGATGAACTAT	DNA/RNA hybrid substrate for RNase H assay
target $D_9R_2D_{16}$	GGATTACACAUGGCATGGATGAACTAT	DNA/RNA hybrid substrate for RNase H assay
target $D_8R_4D_{15}$	GGATTACACAUGGCATGGATGAACTAT	DNA/RNA hybrid substrate for RNase H assay
target $D_9R_4D_{14}$	GGATTACACAUGGCATGGATGAACTAT	DNA/RNA hybrid substrate for RNase H assay
ASO D_{27}	ATAGTTCATCCATGCCATGTGTAATCC	ASO complementary to the 27 nt DNA substrate and DNA/RNA hybrid substrates
ASO179	TGATGGTCTCCATGTCGCTTG	ASO corresponding to guide strand of TBSV-targeting siRNA 179
ASO209	AAATCTCTTTCTTAGGCCAAA	ASO corresponding to guide strand of TBSV-targeting siRNA 209
ASO3243	ATTCGCCAACTCAACTCTATC	ASO corresponding to guide strand of TBSV-targeting siRNA 3243
ASO3701	AAAAACGCACTGTCTGTACCT	ASO corresponding to guide strand of TBSV-targeting siRNA 3701
ASO3722	TTAGAGACAGTACAATTTATG	ASO corresponding to guide strand of TBSV-targeting siRNA 3722
ASO3939	TTCACTGTTAGCTTGTTCCCT	ASO corresponding to guide strand of TBSV-targeting siRNA 3939
ASO209 MOE4	[2'-O-MOE-rA][2'-O-MOE-rA]ATCTCTTTCTTAGGCCA[2'-O-MOE-rA][2'-O-MOE-rA]	2'-O-methoxyethyl-modified variant of ASO209
ASO209 LNA2	[LNA-A]AATCTCTTTCTTAGGCCAA[LNA-A]	Locked Nucleic Acid-modified variant of ASO209
ASO209 PS2	A*AATCTCTTTCTTAGGCCAA*A	Phosphorothioate-modified variant of ASO209 (partial)
ASO209 PS20	A*A*A*T*C*T*C*T*T*T*C*T*T*A*G*G*C*C*A*A*A	Phosphorothioate-modified variant of ASO209 (full)
T7pro2G	CCCTAATACGACTCACTATAGG	forward primer for the generation of template DNA for *in vitro* transcription of the 5'-terminal fragment of TBSV gRNA
TVGM_JS1r	CCTTAAGTTCCCTTGCCGGC	reverse primer for the generation of template DNA for *in vitro* transcription of the 5'-terminal fragment of TBSV gRNA
T7GFP2f	CGTAATACGACTCACTATAGGAGAATCGAGTTAAAAGGTATTG	forward primer for the generation of template DNA for *in vitro* transcription of a fragment of GFP mRNA
TraGFP1r	ATTTGCGGCCGCTTATCTAGATCCGGTGGATCCCG	reverse primer for the generation of template DNA for *in vitro* transcription of a fragment of GFP mRNA
15.22 3´RT	ATTGATGGTGCCTAC	cDNA synthesis of 5' cleavage products (after ligation of the Universal miRNA cloning linker)
GFP2f RACE	AGAATCGAGTTAAAAGGTATTG	forward primer for PCR amplification of 5' cleavage products of GFP target RNA
17.92 3´PCR	ATTGATGGTGCCTACAG	reverse primer for PCR amplification of 5' cleavage products

[a] ribonucleotides are indicated in red

Supplementary Table S2. RNA oligonucleotides used in this study

oligonucleotide	sequence	description
siR gf698 gs	uaguucauccaugccaugugu	guide strand of siRNA gf698 targeting the mRNA of Green Fluorescent Protein (GFP)
siR gf698 ps	acauggcauggaugaacuaua	passenger strand of siRNA gf698 targeting the mRNA of Green Fluorescent Protein (GFP)
siR179 gs	ugauggucuccaugucgcuug	guide strand of siRNA 179 targeting TBSV gRNA
siR179 ps	agcgacauggagaccaucaag	passenger strand of siRNA 179 targeting TBSV gRNA
siR209 gs	aaaucucuuucuuaggccaaa	guide strand of siRNA 209 targeting TBSV gRNA
siR209 ps	uggccuaagaaagagauuuuu	passenger strand of siRNA 209 targeting TBSV gRNA
siR3243 gs	auucgccaacucaacucuauc	guide strand of siRNA 3243 targeting TBSV gRNA
siR3243 ps	uagaguugaguuggcgaauua	passenger strand of siRNA 3243 targeting TBSV gRNA
siR3701 gs	aaaaacgcacugucuguaccu	guide strand of siRNA 3701 targeting TBSV gRNA
siR3701 ps	guacagacagugcguuuuuca	passenger strand of siRNA 3701 targeting TBSV gRNA
siR3722 gs	uuagagacaguacaauuuaug	guide strand of siRNA 3722 targeting TBSV gRNA
siR3722 ps	uaaauuguacugucucuaacc	passenger strand of siRNA 3722 targeting TBSV gRNA
siR3939 gs	uucacuguuagcuuguucccu	guide strand of siRNA 3939 targeting TBSV gRNA
siR3939 gs	ggaacaagcuaacagugaacg	passenger strand of siRNA 3939 targeting TBSV gRNA
